# Magnetic resonance imaging and endorectal ultrasound for diagnosis of rectal lesions

**DOI:** 10.1186/s40001-014-0078-0

**Published:** 2015-01-14

**Authors:** Franciszek Burdan, Iwona Sudol-Szopinska, Elzbieta Staroslawska, Malgorzata Kolodziejczak, Robert Klepacz, Agnieszka Mocarska, Marek Caban, Iwonna Zelazowska-Cieslinska, Justyna Szumilo

**Affiliations:** St. John’s Cancer Centre, 7 Jaczewskiego Str., 20-090 Lublin, Poland; Department of Human Anatomy, Medical University of Lublin, 4 Jaczewskiego Str., 20-090 Lublin, Poland; Department of Radiology, Institute of Rheumatology, 1 Spartanska Str., 02-637 Warsaw, Poland; Department of Diagnostic Imaging, Second Faculty of Medicine, Medical University of Warsaw, 8 Kondratowicza Str., 03-242 Warsaw, Poland; Department of Proctology, Hospital at Solec, 93 Solec Str., 00-382 Warsaw, Poland; Department of Clinical Pathomorphology, Medical University of Lublin, 1 Ceramiczna Str., 20-059 Lublin, Poland

**Keywords:** Endorectal ultrasonography, Magnetic resonance, Perianal fistula, Rectal cancer, Anal cancer

## Abstract

Endorectal ultrasonography (ERUS) and magnetic resonance imaging (MRI) allow exploring the morphology of the rectum in detail. Use of such data, especially assessment of the rectal wall, is an important tool for ascertaining the perianal fistula localization as well as stage of the cancer and planning it appropriate treatment, as stage T3 tumors are usually treated with neoadjuvant therapy, whereas T2 tumors are initially managed surgically. The only advantage of ERUS over MRI is the possibility of assessing T1 tumors that could be treated by transanal endoscopic microsurgery. However, MRI is better for visualizing most radiological prognostic features in rectal or anal cancer such as a circumferential resection margin less than 1 mm, T stage at T1-T2 or T3 tumors with extramural extension less than 5 mm, absence of extramural vascular invasion, N stage at N0/N1, and tumors located in the middle or upper third of the rectum. It can also evaluate the intersphincteric space or levator ani muscle involvement. Increased signal on diffusion weighted imaging (DWI) and low apparent diffusion coefficient (ADC) values as well as an irregular contour and heterogeneous internal signal intensity seem to predict the involvement of pelvic lymphatic nodes better than their size alone. Computed tomography as well as other examination techniques, including digital rectal examination, contrast edema, recto- and colonoscopy, are less useful in staging of rectal cancer but still are very important screening tools.

## Review

The rectum, which is a terminal part of large intestine, is anatomically divided into the anus, anal canal and ampulla. The last two parts are clinically separated into the lower, middle and upper third [1,2]. The most inferiorly located part is surrounded by the so-called sphincter anal complex, formed by the smooth internal anal muscle – the direct continuation of the circular layer of the muscularis propria of the rectal ampulla and colon, as well as the more superficially located striated external anal sphincter and puborectal muscles, which belong to the levator ani muscles. The lowest point of the external anal sphincter indicates the upper anal margin (anal verge), which is a principal landmark for all other rectal measurements. The pectinate (dentate) line is located 1.5-2 cm upwards from the anus. It separates the anal canal into anatomical and surgical parts located below and above the line. The surgical canal (3–4 cm, shorter in females) extends to the anorectal ring/junction, visible at the level of the puborectal sling (lower margin of rectal ampulla). The pectinate line is not visible in any radiological examinations, but below it the inner layer of the rectum is covered with modified skin with the squamous epithelium (anoderm), while above it – like the remaining infradiaphragmatic part of the digestive tract – the mucosa is covered with columnar epithelium. The junction between them is lined with a modified transitional epithelium. It also has to be pointed out that the anoderm is almost directly attached to the internal anal sphincter, since the submucosal layer of connective tissue does not exist on this level. Outside of the rectum, between the middle part of the organ and upper surface of the levator ani, a loose connective tissue known as the mesorectum is located. It contains lymph nodes and neurovascular bundles as well as fat and fibrous tissue. It is limited posterolaterally by the pelvic visceral fascia and ventrally by an upper continuation of the rectogenital membrane (Denonvilliers’ fascia), which extends from the dorsal surface of the prostate and seminal vesicle or fornix of the vagina. In females and males this dense band forms the rectovaginal septum and rectoprostatic fascia, respectively. Laterally, a tiny but easily discernible structure known as the mesorectal or perirectal visceral fascia is seen [1,3]. It is worth mentioning that the volume of perirectal fat is larger in males and positively correlates with the visceral compartment area, but not with the age, body cross-sectional area and body mass index [4] and, most importantly, with staging of the rectal tumor [5]. The mesorectal fat is limited superiorly by the peritoneum. Its interior peritoneal reflection on the anterior rectal wall forms the border between the middle and upper part of the organ.

In spite of lack of submucosal connective tissue at the level of the anatomical canal, like in the remaining parts of the large and small intestine, the rectum has a multilayer wall that can be examined with both endorectal (endoanal) ultrasonography and magnetic resonance imaging. Both techniques are helpful in establishing the morphology of the rectum, most pathological lesions and local staging of neoplasms. Computed tomography as well as other examination techniques, including digital rectal examination, contrast edema, and recto- and colonoscopy, are less useful but still are very important screening tools (Table 1) [6].

**Table 1:** **Algorithm of the pretreatment elective imaging workup for colon and rectal cancer** [6]

**Colon cancer**		**Rectal cancer**
Biopsy during colonoscopy	Diagnosis	Biopsy and full colonoscopy
Abdominal CT or CT colonography	Location	MR
Abdominal CT	T-stage	MRI (stage T1-T4, including evaluation of the mesorectal fascia), EURS (stage T1)
Abdominal CT	N-stage	MR
Abdominal CT or liver MR chest CT or chest X-ray	M-stage	Abdominal CT or liver MR chest CT or chest X-ray

### Imaging modalities

#### Endorectal ultrasonography

Endorectal ultrasonography (ERUS), particularly with a concomitant examination by the linear transducer, is highly effective in most cases. It is usually performed without any previous preparation, but an enema strongly improves the image quality, especially when there are stool residues in oncological patients [7].

**Figure 1:**
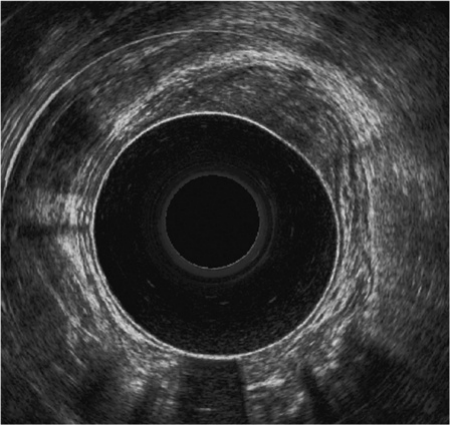
**Rectal endosonography with a water balloon.** A tumor limited to the mucosa, not invading the submucosa and muscularis propria.

The examination is performed in the left recumbent (left decubitus, semiprone, Sims) or less frequently in the knee-elbow position, which is preferred in patients with sphincter insufficiency. Ultrasound is usually done after digital rectal examination and proctoscopy, using a mechanical or biplanar transducer with a frequency of 10 MHz or higher [7-14]. Some authors suggest using a 3D 16-MHz probe, which allows spatial analysis of both the rectum and surrounding structures including muscles and mesorectal fat. This system also permits archiving the full examination [8]. Higher frequencies give better resolution of the rectal wall and sphincter complex, while lower ones are helpful in a mesorectum examination [15]. Due to the high cost, special water-filled balloons are usually applied only in patients with tumors located in the rectal ampulla (Figure 1). In such cases, the balloon is filled with about 90 ml of water, which compresses the lesion and removes the air from the rectum [7].

Typically in ultrasound images five layers of the rectal wall are visible: three hyper- and two hypoechoic ones [7]. The inner hyperechoic layer represents the interface between the probe/covering balloon and the mucosa. The second layer – the hypoechoic one – indicates the mucosa, muscularis mucosa and submucosa, which cannot be differentiated sonographically. More superficially, there is another hyperechoic line corresponding to the interface between the submucosa and muscularis propria (proper muscular layer of the rectum indicated by the next hypoechoic line). The most external hyperechoic line corresponds to the interface between the muscularis propria and perirectal fat or visceral layer of the peritoneum in the upper part of the ampulla [16]. Beynon et al. [17] suggests that the mucosa and submucosa can be distinguished, and the second layer (the hypoechoic one) is formed exclusively by the mucosa and muscularis mucosa, while the third hyperechoic line corresponds to the submucosa.

**Figure 2:**
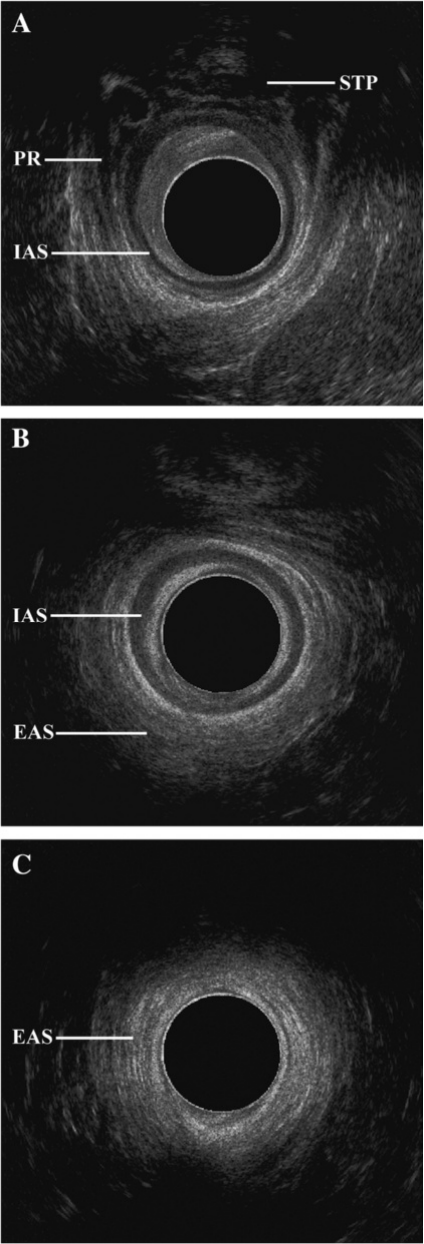
**Endosonographic morphology of the anal canal: a high level (A) at the level of the sling of the puborectalis muscles (PR) and superficial transverse perineal muscles (STP), mid level (B) with a well-formed internal (IAS) and external anal sphincter (EAS), and superficial/low level (C) with the external anal sphincter.**

Depending on the position of the probe, the surrounding muscles could also be seen. In the low third of the rectum (at the level of the anal canal), usually the external anal sphincter should be visible (Figure 2). Slightly above it is replaced by the para-, recto- and prerectal fibers of the levator ani, which at this level is formed by the puborectal muscle. Between the puborectal sling and caudally located external sphincter, there is an intersphincteric plane filled with the lowest, tapered part of the mesorectum [18]. This narrow layer is important in planning any surgical procedures at the level of the anal canal and for the staging of lower anal cancer. Moreover, the muscle divides the anal canal into the upper and the lower part. In front and slightly below the midrectal level, the urogenital hiatus is limited posteriorly by the superficial transverse perineal muscle. Above, the seminal vesicles, prostate, urinary bladder, and urethra or vagina and uterus can also be observed in males and females, respectively. Irrespective of gender, loops of the small intestine and occasionally a low-positioned vermiform appendix may be seen as well [15].

In case of perianal fistulas, a hyperechoic contrast agent (e.g., 3% hydrogen peroxide solution) injected directly into the pathological canal is recommended [8]. However, typical intravenous sonographic contrast agents are seldom used, mainly because of the high cost but also because of the relatively short time required for picture acquisition.

The main limitation of ERUS application is tumors located close to the sigmoid colon or infiltrating adjacent organs. Moreover, it is a gold standard initial examination for fecal incontinence [13], but an anorectal manometry is also recommended in such cases [19-23].

### Magnetic resonance

Magnetic resonance imaging (MRI), with both endorectal and pelvic phased array coils, is the gold standard, especially in oncological rectal examination. An endorectal coil is a surface coil and provides very good images of the wall of the organ, but offers limited information on surrounding structures. Moreover, the best images are taken only at the level of the coil. Its usefulness is also limited because of the patient discomfort and in case of anal stenosis. Rectal wall motion is also responsible for the coil migration and misinterpretation of the observed lesions. For these reasons phased array coils are recommended in routine cancer staging. They obtain higher signal, but with wider coverage and improved homogeneity [24]. In rectal cancer, the pelvic coil showed high accuracy in tumor (59-95%) and nodal metastasis evaluation (39-95%). Similar data for the endorectal coil reached 66–91 and 72-79%, respectively [25,26], and for T staging was similar to ERUS [27,28]. However, it has to be stressed that unlike for ultrasound, there are a number of absolute and relative contraindications for MRI (Table 2). The examination can be performed in pregnant women, but without contrast media administration and using a limited number of sequences [29,30].

**Table 2:** **Contraindications for magnetic resonance imaging** [30]

**Absolute**	**Relative**
- Pacemaker	- Pregnancy
- Cochlear implants	- Claustrophobia
- Metallic object in the eye ball	- Metal objects in soft tissues
- No verbal contact with patients (deafness)	- Metal orthopedic treatment elements
	- Prosthetic cardiac valve
	- Dental implants
	- Monitoring/dosing devices
	- Intrauterine device
	- Permanent makeup
	- Tattoo

The examination does not require any previous rectal preparation but in some medical centers a prior enema is recommended. Furthermore, the usage of intrarectal water, ultrasound gel, gadolinium or air insufflations as well as pretreatment with spasmolytic agents is debatable, but they may strongly improve image quality [2]. Most skeptics stress that any kind of artificial substances inside the rectum may be uncomfortable to the patient and, more importantly, may compress the mesorectal fat and influence evaluation of the circumferential resection margin. The main exception to the contrast enema is a dynamic rectal examination, also known as MRI defecography or proctography, performed for various pelvic floor dysfunctions [31]. However, similar data, such as for Park’s angle and various mobile rectal diameters, could be obtained during ERUS and ultrasound examination of the perineum with a linear probe [7,13].

The rectal MRI examination is performed on 1.5-T or higher systems, but the reported sensitivity and specificity for 3 T are very similar to those obtained for 1.5 T. However, due to the high signal, 3-T equipment may obtain thinner T2-weighted images, which are the most suitable for rectal evaluation [32].

**Figure 3:**
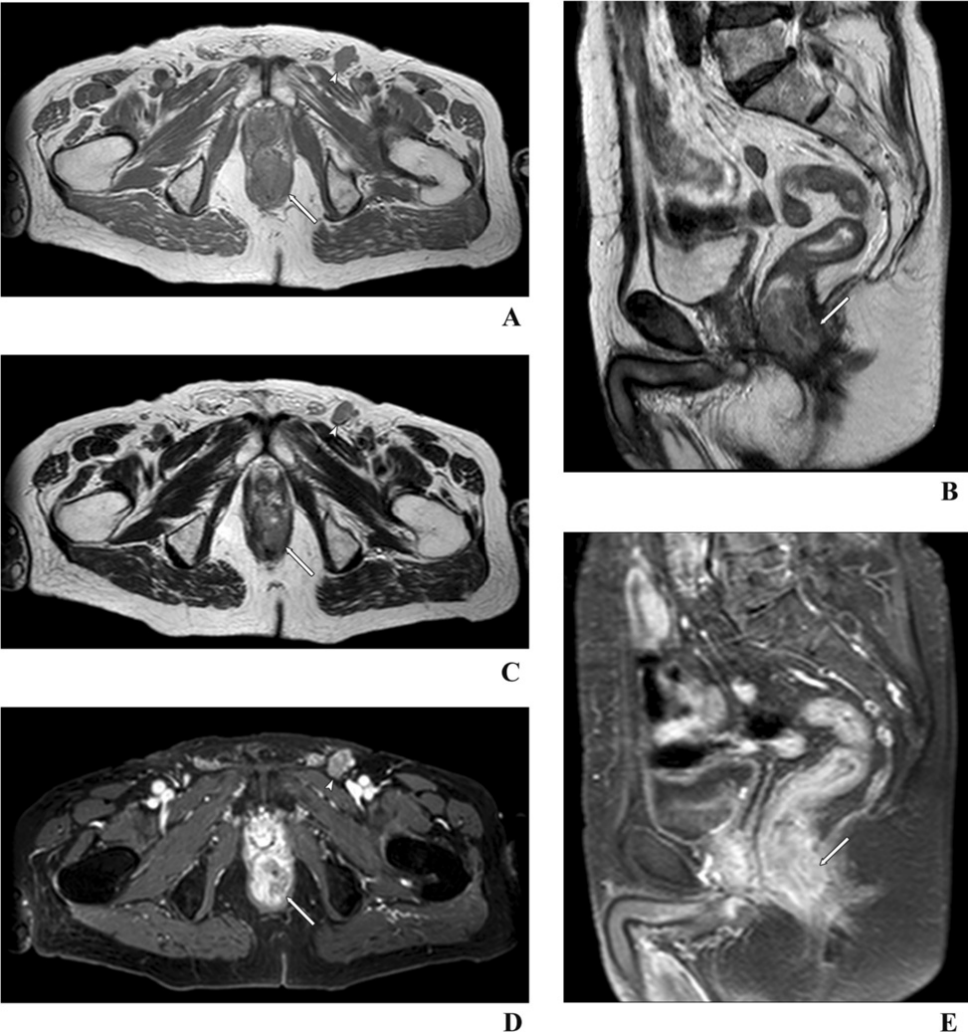
**Anal cancer with invasion of the intersphincteric space, external anal sphincter (arrow) and metastasis in the inguinal lymph node (arrowhead).** Examination with a 1.5-T pelvic phased array coil. Axial T1- **(A)**, T2- **(C)** and T1-weighted with fat suppression post-gadolinium-enhanced images **(D)**. Sagittal T2- **(B)** and T1-weighted images with fat suppression post-gadolinium-enhanced images **(E)**.

**Figure 4:**
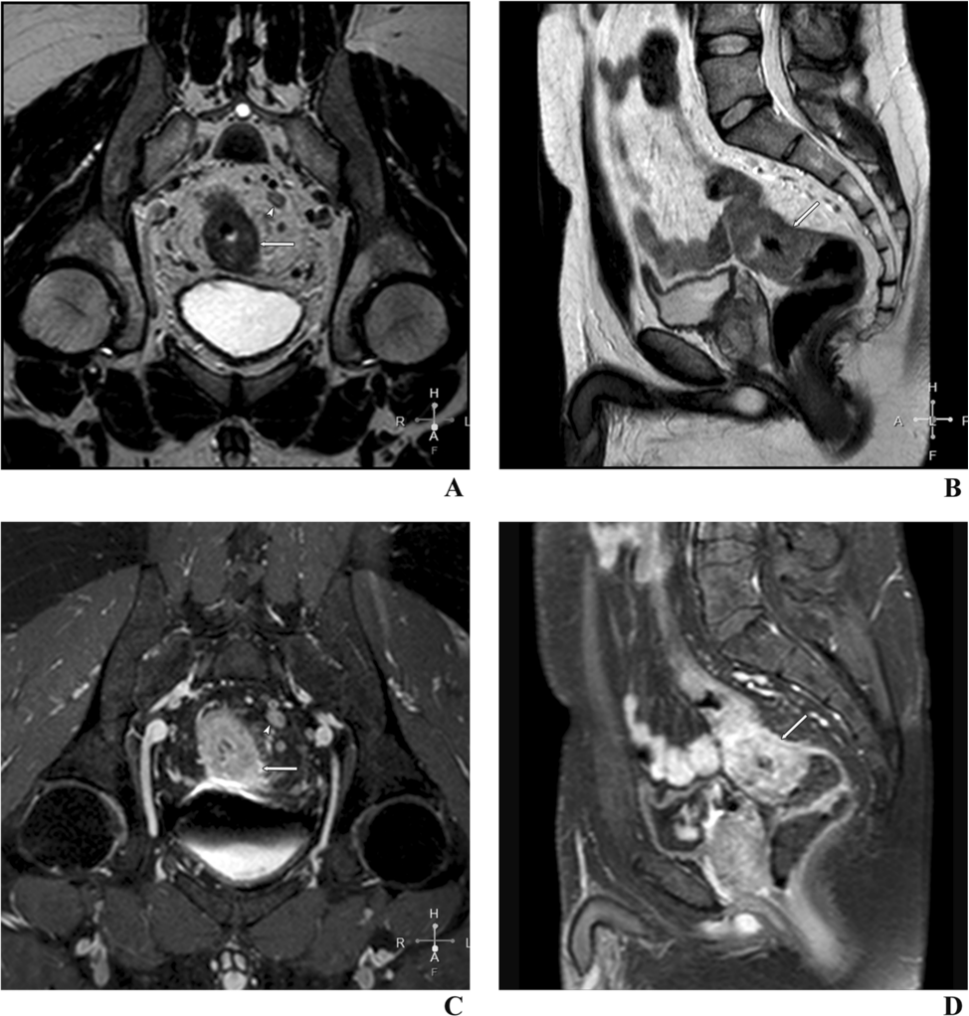
**Rectal cancer with extramural extension in perirectal fat (arrow) and metastasis in a lymph node (arrowhead).** Examination with a 1.5-T pelvic phased array coil. Oblique T2- **(A)** and T1-weighted with fat suppression post-gadolinium-enhanced images **(C)**. Sagittal T2- **(B)** and T1-weighted with fat suppression post-gadolinium-enhanced images **(D)**.

**Figure 5:**
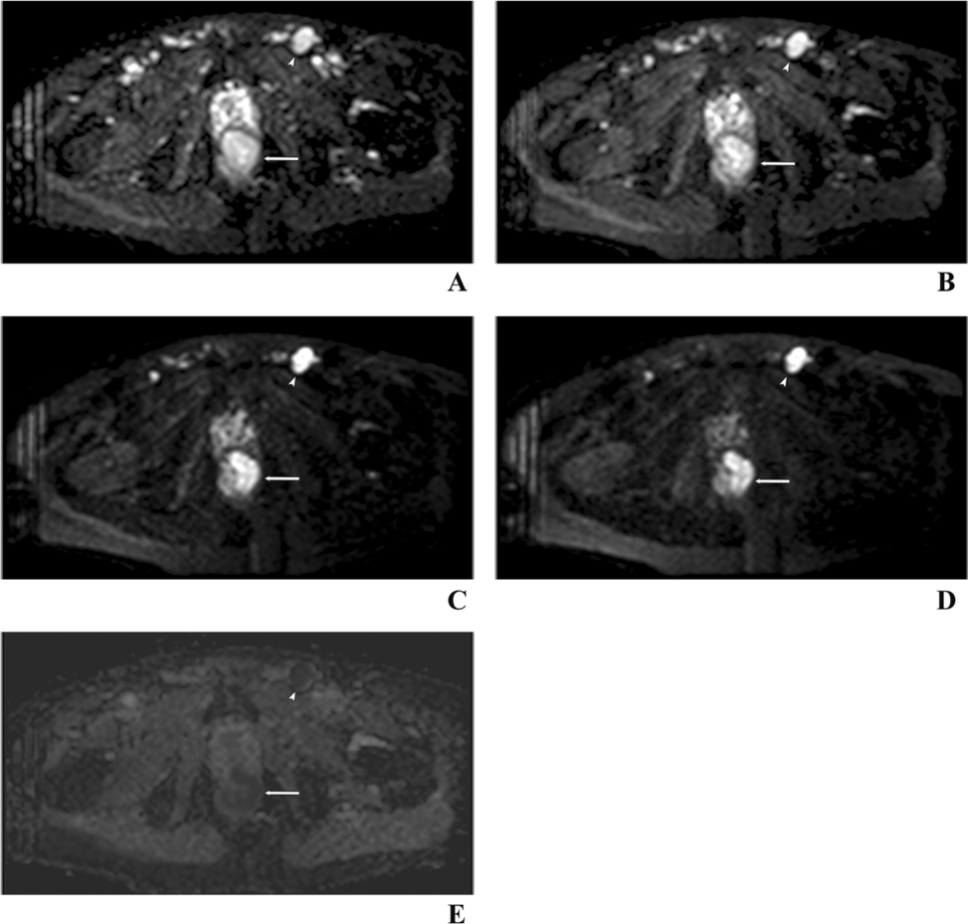
**Anal cancer (arrow) and metastasis in an inguinal lymph node (arrowhead) in the same patient as in Figure** 3 **.** Examination with a 1.5-T pelvic phased array coil. DWI images at b = 0 **(A)**, 50 **(B)**, 500 **(C)** and 1,000 s/mm^2^ **(D)** as well as the corresponding apparent diffusion coefficient (ADC) map **(E)** at b = 1,000 s/mm^2^. The ADC value for the tumor and lymph node 0.867 × 10^−3^ and 0.809 × 10^−3^ mm^2^/s, respectively.

During the examination, the patient has to be comfortably positioned in a supine position, since image acquisition usually takes about 40–60 min. Like in most pelvic examinations, breath holding is not recommended. After initial coronal and sagittal localization images, usually a sagittal T2-weighted, fast (turbo) spin-echo sequence is performed. It is followed by large-field-of-view axial sections of the whole pelvis and T2-weighted thin-section axial sections perpendicular to the long axis of the rectum (Tables 3 and 4; Figures 3 and 4). In case of low rectal cancers, high-spatial-resolution coronal imaging is recommended just to see their position in relation to the levator ani and sphincter complex [33]. It is especially important in planning sphincter-sparing surgery [34]. In perianal fistulas, small-field-of-view axial T2-weighted sections seem to be more reasonable. Moreover, radial water- and fat-saturation sequences are useful to obtain spatial pseudo 3D reconstruction. Such MRI hydrography has been previously used routinely to visualize the biliary and pancreatic ducts (MRCP) and urinary pathways through depiction of static fluid. Currently, in most medical centers the examination is performed according to the suggestions of The Magnetic Resonance Imaging and Rectal Cancer European Equivalence Study (MERCURY) [35]. Three-dimensional (3D) T2-weighted sequences permit application of 1–2-mm-thin sections with no intersection gap. They should be able to compensate for difficulties with angulation of the tumor such as tortuosity and redundancy of the rectum [18]. Usefulness of the gadolinium contrast enhancement is debatable, since it does not improve evaluation of local staging [36]. Moreover, since contrast enhancement requires fat suppression, it results in reduction of the signal-to-noise ratio and potential overstaging of the tumor due to enhancement of adjacent nonmalignant structures such as the vessels, desmoplastic stromal reaction and normal nodes [24]. Unlike other authors, Zhang et al. [34] indicated that 3D fat-suppressed dynamic contrast enhancement is the best technique to delineate the tumor margins. On the other hand, contrast injection may be helpful in examination of the internal morphology of the tumor as well as various perfusion values, which are important in the evaluation of an early treatment response and in tumor recurrence. For the same reason, diffusion-weighted images (DWIs) should be routinely performed, using increasing b values (in our hospital usually 0, 50, 500 and 1,000 s/mm^2^) (Figure 5). Even though the DWI images together with ADC maps are very helpful in detection of the lesions, they cannot be applied to confirm malignancy without examination of classical T1- and T2-weighted sequences [37]. In both perfusion and ADC measurements, the region of interest (ROI) should only fit the pathological lesion, without the neighboring tissues. Even ADC measurement might be subjective; Attenberger et al. [38] indicated that by using strict criteria they could be analyzed with good interobserver agreement in patients with rectal cancer.

**Table 3:** **Characteristics of standard sequences in MRI rectal examinations** [24]

	**Philips**	**Siemens**	**General Electric**
	**(Turbo spin echo)**	**(Turbo spin echo)**	**(Fast spin echo)**
Repetition time (ms)	5,080 (sagittal)	3,000-6,000	4,000
	4,018 (axial)		
Echo time (ms)	132 (sagittal)	100	110
	80 (axial)		
No. of slices	23 (20 axial)	24	24
Thickness/gap	3 (sagittal)	5/0	5/0
	5/1 (axial)		
Interleaved	No	Yes	NO
Echo train length	23	8	8
Matrix in phase direction	512	512	512
Matrix in frequency direction	370/70% (sagittal)	256	288
	256/100% (axial)		
Phase encoding direction	AP	AP	AP
Field of view (mm)	250	250	250
Phase	250	240	250
Frequency	250	240	250
No. of acquisitions	3 (sagittal)	2	2
	2 (axial)		
Flow compensation	Yes	Yes	Yes
Sat bands	Anterior/superior	Anterior	Anterior

**Table 4:** **Characteristics of standard sequences in MRI rectal examinations – continuation** [24]

	**Philips**	**Siemens**	**General Electric**
	**(Turbo spin echo)**	**(Turbo spin echo)**	**(Fast spin echo XL)**
Repetition time (ms)	5,362	6,590	5,100
Echo time (ms)	100	136	85
No. of slices	16	24	28
Thickness/gap	3/0.3	3	3
Interleaved	Yes	Yes	No
Echo train length	16	8	8
Matrix in phase encoding	256	256	256
Matrix in frequency encoding	256/90%	256	256
Phase encoding direction	Foot to head	Foot to head	Superior inferior
Field of view (mm)	160	160	160
Rectangular field of view	100%	100%	160
Foldover	Right to left	Right to left	No phase wrap
No. of acquisitions	6	4	4
Sat bands	None	Superior inferior	Superior inferior
Scan duration (min:s)	7:35	7:36	8:40
Other	No DRIVE	No DRIVE	Phase correct on
	Prep phase auto	Prep phase auto	Flow camp on
			Tailored radiofrequency fast

From the clinical point of view, T2-weighted images without fat saturation are the most helpful in distinguishing normal morphology of the rectal wall from organ abnormalities. The most internal hyperintense layer is formed by the mucosa and submucosa, which cannot be differentiated by either MRI or ERUS. The next hypo- and hyperintense layers represent the muscularis propria and perirectal fat, respectively. Usually, the mesorectal fascia should be also detected as a low-signal line visible on the margin of the mesorectum [2]. However, there are studies in which five layers were clearly described. Stollfuss et al. [39] were able to distinguish hypointense mucosa and hyperintense submucosa in 19 of 24 post-resection specimens using T2-weighted images obtained on a 1.5-T system. The internally located circular-directed muscular fibers had higher signal than the outer longitudinal-directed fibers.

It is also important that MRI may differentiate mucinous from nonmucinous adenocarcinomas [40,41] and may be helpful in the identification of early recurrence from postoperative changes (perianal fistulas and sinuses) [11,12]. Moreover, unlike CT, MRI does not use ionizing radiation and highly nephrotoxic contrasts.

### Description of rectal lesions in ERUS and MR

In the final radiological report, any abnormalities should be identified and clearly described. In both malignant and nonmalignant rectal lesions various calcifications have been found. Anatomically or for general description, rectal lesions, like the organ itself – as described in detail in the introduction – may be divided into lower, middle and upper ones. However, in clinical practice, the Parks [8,12] and WHO TNM classifications are commonly used (Table 5) [42]. The former describes four primary types of perianal fistulas: intersphincteric, transsphincteric, suprasphincteric and extrasphincteric. The latter is used for tumor staging and helps to divide patients for surgery (stage T1 and T2) or for preoperative therapy (>T2). Nowadays, patients with T1 stage undergo transanal endoscopic microsurgery, while those with stages T2 and T3 are usually treated with a total mesorectal excision, without or after preoperative neoadjuvant therapy (radio- or radiochemotherapy), respectively. Such a therapeutic schema highly increases the 5-year survival rate when compared with conventional surgery. Moreover, patients with preoperative neoadjuvant therapy had a substantially lower rate of local recurrence compared with those who received similar postoperative treatment [43]. On the other hand, low rectal tumors form a distinct entity among all neoplasms of the organ as they have a high risk of local recurrence and poor outcome compared to lesions located in the middle and upper rectum [44]. It is especially important in the abdominoperineal excision, which is characterized by a relatively high risk of local recurrence (>30%) [35].

**Table 5:** **TNM staging principles for the most common rectal neoplasms: anal (anal canal) and rectal (ampulla) carcinoma, carcinoid and gastrointestinal stromal tumors (GIST) according to the current classification of International Agency for Research on Cancer/World Health Organization** [42]

	**Anal carcinoma**	**Rectal carcinoma**	**Carcinoid of the rectum**	**GIST**
T	Primary tumor			
TX	Primary tumor cannot be assessed	Primary tumor cannot be assessed	Primary tumor cannot be assessed	Primary tumor cannot be assessed
T0	No evidence of primary tumor	No evidence of primary tumor	No evidence of primary tumor	No evidence of primary tumor
Tis	Carcinoma in situ, Bowen disease, high-grade squamous interepithelial lesion (HSIL), anal interepithelial neoplasia II-III (AIN II-III)	Carcinoma in situ: intraepithelial or invasion of lamina propria		
T1	Tumor 2 cm or less in the greatest dimension	Tumor invades submucosa	Tumor invades lamina propria or submucosa and is no greater than 2 cm in size	Tumor 2 cm or less in greater dimension
			T1a – tumor less than 1 cm in size	
			T1b – tumor 1 to 2 cm in size	
T2	Tumor more than 2 cm but not more than 5 cm in the greatest dimension	Tumor invades muscularis propria	Tumor invades muscularis propria or is greater than 2 cm in size	Tumor more than 2 cm but not more than 5 cm
T3	Tumor more than 5 cm in the greatest dimension	Tumor invades subserosa or into non-peritonealized perirectal tissues	Tumor invades subserosa or non-peritonealized perirectal tissues	Tumor more than 5 cm but not more than 10 cm
T4	Tumor of any size invades adjacent organ(s), e.g., vagina, urethra, bladder (direct invasion of rectal wall, perianal skin, subcutaneous tissue or the sphincter muscle(s) alone is not classified as T4)	Tumor perforates visceral peritoneum (T4a) and/or directly invades other organs or structures (T4b)	Tumor perforates peritoneum or invades other organs	Tumor more than 10 cm in the greatest dimension
N	Regional lymph nodes			
NX	Regional lymph nodes cannot be assessed	Regional lymph nodes cannot be assessed	Regional lymph nodes cannot be assessed	Regional lymph nodes cannot be assessed
N0	No regional lymph nodes metastasis	No regional lymph nodes metastasis	No regional lymph node metastasis	No regional lymph nodes metastasis
N1	Metastasis in perirectal lymph nodes	Metastasis in 1 to 3 regional lymph nodes	Regional lymph node metastasis	Regional lymph node metastasis
		N1a - Metastasis in 1 regional lymph node		
		N1b – Metastasis in 2 to 3 regional lymph nodes		
		N1c Tumor deposit(s), i.e. satellites, in the subserosa or in non-peritonalized pericolic or perirectal soft tissue without regional lymph node metastasis		
N2	Metastasis in unilateral internal iliac and/or inguinal lymph nodes	Metastasis in 4 or more regional lymph nodes		
		N2a - metastasis in 4 to 6 more regional lymph nodes		
		N2a - metastasis in 7 or more regional lymph nodes		
N3	Metastasis in perirectal and inguinal lymph nodes and/or bilateral internal iliac and/or bilateral inguinal lymph nodes			
M	Distal metastasis			
M0	No distal metastasis	No distal metastasis	No distal metastasis	No distal metastasis
M1	Distal metastasis	Distal metastasis	Distal metastasis	Distal metastasis
		M1a – metastasis confined to one organ		
		M1b – metastasis in more than one organ or the peritoneum		

In nonmalignant lesions ERUS offers similar accuracy to MRI and could be easily applied in all pathologies located close to the rectal wall. Due to the higher spatial resolution, it is even better than MRI in differentiating T1 (T1sm1 and T1sm2) and T2 tumors, but is more subjective and depends highly on the sonographer’s experience [45]. In contrast, MRI is more reproducible and allows accurate evaluation of neoplastic and distal spread, including measurement of mesorectal involvement and establishing the potential surgical circumferential resection margin. According to Wieder et al. [46], MRI led to accurate prediction of the circumferential resection margin with 100% sensitivity and 88% specificity, which depend on the minimum distance of the tumor to the mesorectal fascia seen in histological examinations. It is also a method of choice to exclude infiltration of structures located nearby, but in such an examination the gadolinium contrast enhancement strongly increases the accuracy [18,46,47]. The main problem with MRI is an overstaging usually caused by a desmoplastic reaction in nonneoplastic structures located close to the tumor margin [47]. It usually happens in case of stage T2 and T3. However, in stage T3 the muscularis propria signal intensity is unclear, while its external margin loses smoothness and becomes more nodular or spiculated. On the other hand, hyperplasia of the muscularis propria is associated with radiation and may also increase overinterpretation [39]. It is worth mentioning that computed tomography (CT) is not indicated in anal tumor diagnosis because of low sensitivity (66%) [48,49].

Irrespective of the applied method for each tumor, clinicians require the following information: tumor spread, including detailed evaluation of the mesorectal fascia (T feature) and extramural venous invasion, lymph node involvement (N feature) and presence of distal metastases (M feature). However, in case of rectal abnormalities the distance from the sphincter anal complex as well as the sphincters’ morphology and their function are also important because a low rectal tumor without any sphincter invasion and a distance between its inferior pole and the upper margin of the internal sphincter may be treated with low anterior resection consisting of en-bloc resection of the rectum with total mesorectal excision [18]. If possible, the distance to the mesorectal fascia or levator ani muscles for low rectal tumors should also be established. From a clinical point of view, it is more important to measure the depth of extramural spread in the mesorectal fat than to ascertain the T stage, since a T2 tumor has the same prognosis as a T3 tumor with less than 1 mm spread. Moreover, T3 tumors with less and more than 5 mm mesorectal invasion have different 5-year survival rates, i.e., 85 and 54%, respectively [50,51]. According to Beets-Tan et al. [52], perirectal spread terminating at least 5 mm from the fascia predicts an uninvolved circumferential margin of 1 mm at histological analysis with 97% confidence. However, such measurement is difficult and imprecise in tumors located in a lower third of the anal canal, on its anterior wall or in patients with a small amount of perirectal fat [2]. All these features are important prognostic factors and crucial for therapeutic management.

Extramural venous invasion – beyond the muscularis propria in an endothelium-lined vessel – is important since neoplastic cell embolism in the portal circulation may initiate distant metastases through hematogenous spread [53]. It is associated with a higher incidence of local and distant metastases as well as poorer overall survival rates [54,55]. Such complications were observed histopathologically in 10-54% cases of rectal cancer [56], but could be also evaluated by MRI but not CT [57]. A recent report [58] states that patients with MRI-detected venous invasion had a 3.7 times increased relative risk of metachronous metastatic disease. The most common system (Table 6) applied for such evaluations was introduced by Smith et al. [59] and divides the tumors into lesions without (score 0–2) and with venous invasion (score 3–4). Additionally, each vessel should be described as a small (a perforating vein that runs perpendicular to the rectal lumen), medium (an unnamed vein that runs parallel to the rectal lumen) or large named vein [58]. It is worth mentioning that in extramural venous invasion an abdominal MRI follow-up is recommended, since according to Scharitzer at al. [60], a gadoxetic-acid-enhanced 3-T system is more sensitive than 64-row multidetector CT in the detection of small (≤10 mm) hepatic metastases. On the other hand, MRI’s usefulness, especially in minute lesions, is limited by various artifacts related to the patient’s condition (e.g., respiratory and cardiac motion, vascular pulsation, nearby located small cysts, liver steatosis and iron accumulation, etc.) as well as artifacts and pitfalls related to the scanner and/or magnetic elements inside of the examined patient (i.e., aliasing, black boundary, chemical shift, entry slice phenomenon, Gibbs energy, magnetic susceptibility, moiré fringes, RF overflow, shading, slice-overlap, susceptibility, zebra stripes, zippers) [30,61,62]. This examination technique is also limited because of its cost and insufficient availability in many institutions. However, according to the algorithm of the European Registration of Cancer Care (EURECCA) [6], the M staging includes MRI or CT examination of the abdomen (Table 1). Furthermore, for detailed lung evaluation only CT is recommended [63]. The usefulness of PET-CT is limited only to multivisceral metastases and differentiating between fibrosis and tumor, particularly in locally recurrent rectal cancer. However, in such cases the DWI significantly improves the diagnosis [64].

**Table 6:** **Extramural vascular invasion scoring system by Smith et al.** [59]

Score 1	Tumor extension through the muscle layer is not nodular, lack of vessels adjacent to areas of tumor penetration
Score 2	Minimal extramural stranding/nodular extension, but not in the vicinity of any vessels
Score 3	Extramural vessels adjusted to the tumor, but these vessels are of normal caliber, and there is no definite tumor signal within the vessel
Score 4	Intermediate signal intensity apparent within the vessels, although the contour and caliber of these vessels are only slightly expanded
Score 5	Obvious irregular vessel contour or nodular expansion of the vessel by definite tumor signal

Nowadays, the main problem in rectal cancer staging is evaluation of lymphatic spread. The difficulty lies in the lack of proper radiological criteria for nodal metastatic changes in the pelvis and various lymphatic pathways that carry the lymph from the rectum [1]. The lymph from the upper part of the ampulla is usually drained via pararectal lymph nodes located on the muscularis propria, either through sacral lymph nodes or directly into inferior mesenteric lymph nodes along the inferior rectal vessels. The remaining part of the rectum over the pectinate line drains the lymph directly to the sacral lymph nodes or via vessels surrounding the middle rectal artery into the internal iliac lymph nodes. All those nodes may not be visible in physiological conditions in ERUS and MR. The inferior third of the rectum, below the pectinate line, sends the lymph into the horizontal part of the superficial lymph nodes that are clearly visible in most radiological procedures.

For the evaluation of lymphatic spread, a pelvic phased array coil is recommended. Like CT, it gives an opportunity to examine most regional lymph nodes [6,35]. Discrimination between normal and metastatic lymph nodes by MRI remains problematic. For a very long time, the CT criteria that stress the lack of an oval shape and fatty sinus, round or irregular margin, as well as short transversal diameter over 10 mm, were applied to MR. However, while using such criteria, the reported accuracy was relatively low and reached only 43-85% [65]. Some authors described even lower thresholds for pararectal lymph nodes, but the sensitivity, specificity and accuracy in lymph node metastases with diameters greater than 6 mm were only 57, 88 and 76%, respectively [66]. Using a 5-mm short axis as a threshold resulted in lower sensitivity (66%) and specificity (76%) [67]. Currently, there is a tendency to report any pelvic lymph nodes since their transversal diameters have not been established in any large and multicenter studies. The irregular borders and signal intensity are principle features in node metastasis and allow much higher sensitivity (85-95%) and specificity (95-97%) [68]. Nowadays, according to the EURECCA principles, nodes >3 mm can be characterized as malignant or benign by signal and border features [6]. On high-resolution MR, identification of nodes <3 mm in diameter containing metastatic foci remains a challenge. Furthermore, most publications stress that the presence of ≥4 lymph nodes is associated with a higher risk of local recurrence [69]. However, based on new clinical observations [70,71], a well-preformed total mesorectal excision limiting nodal involvement is no longer a risk factor for a local recurrence. On the other hand, identification of involved lymph nodes located outside of the mesorectal fascia is important, as they will not be removed during a standard anterior rectal resection with total mesorectal excision [18]. Such nodes may require additional treatment since they are responsible for local recurrence.

In a problematic situation, high signal in DWI and low on ADC maps, as well as low ADC values, could also be helpful, since in metastatic infiltration similar features are seen for the tumor and nodes. However, the main limitation of the method is the relatively large size of the ROI (≥1 cm^2^) and at least three or four different b values used during the acquisition of the DWI sequence [30]. Attenberger et al. [38] found that ADC measurements and functional parameters were useful in differentiating N stages. However, Mizukami et al. [72] reported a high negative predictive value of DWI; even positive nodes had at least 1-cm diameter in the short axis. Promising data came from studies with new contrast agents (i.e., ferumoxtran-10, USPIO), but they are not routinely applied in clinical practice, and most of them are not registered for daily radiological practice [73,74]. Moreover, Lambregts et al. [75] introduced a novel method utilizing gadofosveset enhancement to assess chemical-shift artifacts associated with lymph node involvement, but data have not been confirmed in a multicenter study.

On the other hand, it has to be pointed out that Bipat et al. [76] indicated that in lymph node involvement and organ invasion, the estimated sensitivity and specificity of ERUS, MRI and CT were similar. However, in muscularis propria invasion, ERUS and MRI imaging had similar sensitivities, but the specificity of sonography was significantly higher (86 vs. 69%). Similar data were established for mesorectal invasion. The sensitivity of ERUS (90%) was higher than for CT (79%) and MRI (82%), while the specificities were comparable. In nodal metastatic spreading, the opposite data were recently presented by Puli et al. [77], who concluded that the lower accuracy of EURS in comparison to MRI and CT is due to lack of visualization of the entire mesorectum.

## Conclusion

Endorectal ultrasonography (ERUS) and magnetic resonance imaging (MRI) have become the state of the art in radiological examination of the terminal part of the digestive tract. Both techniques allow a detailed evaluation of the multilayer wall of the rectum, which is obligatory in ascertaining the stage of the rectal and anal cancer and therapeutic strategy, as tumors in stage T2 and T3/T4 are usually treated surgically or with neoadjuvant therapy, respectively. The only advantage of ERUS over MRI is the possibility of assessing T1 tumors that could be managed by transanal endoscopic microsurgery. However, MRI is more precise in visualizing the perirectal fat, mesorectal fascia and peritoneal involvement, extramural venous invasion as well as surrounding organ infiltration. It may also evaluate an involvement of the intersphincteric space or levator ani muscles. In spite of well-established criteria for local tumor spreading, there are no proper principles for lymph node involvement. Increased signal on DWI and low ADC values as well as irregular contour and heterogeneous internal signal intensity seem to predict the involvement of pelvic lymphatic nodes better than their size alone. Because of the lack of any radiological principles for pelvic nodular spreading, the European Registration of Cancer Care suggests relying on MRI for the T-substage, mesorectal fascial involvement and extramural vascular invasion, as nodal staging is less effective. Based on all the presented data, the following are good prognostic features in rectal cancer: the tumor has a circumferential resection margin less than 1 mm, T stage at T1-T2 or T3 tumors with extramural extension less than 5 mm, absence of extramural vascular invasion, N stage at N0/N1, and tumors located in the middle or upper third of the organ. In low rectal tumors, the lack of compromising of the intersphincteric space or levator ani muscles was also described [18].

## Abbreviations

ADC: apparent diffusion coefficient
CT: computed tomography
DWI: diffusion weighted images
ERUS: endorectal ultrasonography
EURECCA: European Registration of Cancer Care
MERCURY: Magnetic Resonance Imaging and Rectal Cancer European Equivalence Study
MRI: magnetic resonance imaging
MRCP: magnetic resonance cholangiopancreatography
PET: positron emission tomography
ROI: region of interest
TNM: tumor nodal metastasis classification of malignant tumors
WHO: World Health Organization
